# Serum Biomarkers in Patent Ductus Arteriosus in Preterm Infants: A Narrative Review

**DOI:** 10.3390/biomedicines13030670

**Published:** 2025-03-09

**Authors:** Manuela Cucerea, Raluca Marian, Marta Simon, Madalina Anciuc-Crauciuc, Andreea Racean, Andrea Toth, Zsuzsánna Simon-Szabó, Mihaela-Georgiana Fadur, Valeriu Moldovan, Elena Moldovan

**Affiliations:** 1 Neonatology Department, George Emil Palade University of Medicine, Pharmacy, Science and Technology, 540142 Târgu Mures, Romania; manuela.cucerea@umfst.ro (M.C.); marta.simon@umfst.ro (M.S.); madalina.anciuc@umfst.ro (M.A.-C.); andreea.racean@umfst.ro (A.R.); andreanoemi29@gmail.com (A.T.); 2 Cellular and Molecular Biology Department, George Emil Palade University of Medicine, Pharmacy, Science, and Technology, 540142 Targu Mures, Romania; 3 Department of Pathophysiology, George Emil Palade University of Medicine, Pharmacy, Science and Technology of Targu Mures, 540142 Targu Mures, Romania; zsuzsanna.simon-szabo@umfst.ro; 4 Department of Neonatology, Targu Mures County Emergency Clinical Hospital, 540136 Targu Mures, Romania; georgiana_fadur@yahoo.com; 5 Molecular Biology, Clinical Laboratory Targu Mures County Emergency Clinical Hospital, 540136 Targu Mures, Romania; valeriumoldovan@gmail.com; 6 Pediatric Intensive Care Unit, Cardiovascular and Transplant Emergency Institute, 540136 Targu Mures, Romania; bitirelena@gmail.com

**Keywords:** PDA, preterm infant, natriuretic peptides, cardiac troponin T, MR-proADM, endothelin-1, isoprostanes, inflammatory biomarkers

## Abstract

**Background**: Patent ductus arteriosus (PDA) in preterm infants presents a significant challenge in neonatal care, marked by ongoing debates about its definition, diagnosis, treatment options, and effects on patient outcomes. Plasma biomarkers assess mediators involved in PDA closure and hemodynamic responses, assisting in identifying newborns at higher risk of developing potentially serious neonatal conditions. The purpose of this review was to investigate the relationship between PDA and various plasma biomarkers used to evaluate and diagnose ductal patency during perinatal life, as outlined in the relevant literature. **Methods**: We conducted an electronic search of the National Library of Medicine (MEDLINE)/PubMed and Web of Science for relevant studies published up to December 2024, including prospective, retrospective, cohort, and cross-sectional studies, as well as reviews and meta-analyses. The keywords used in the search included “preterm infant”, “persistent ductus arteriosus”, “patent ductus arteriosus”, “PDA”, “neonatal biomarkers”, “cardiac biomarkers”, and “vasoactive biomarkers”. **Results**: Out of the 813 identified articles, 85 were included in our review of cardiac biomarkers: Natriuretic peptides (NPs), Cardiac troponin T (cTnT), vasoactive biomarkers (Mid-regional pro-adrenomedullin (MR-proADM), Endothelin-1 (ET-1), Copeptin, and Isoprostanes (IPs)), and inflammatory biomarkers (Interleukin-6 (IL-6), IL-8, IL-10, Growth Differentiation Factor 15 (GDF-15), Monocyte Chemoattractant Protein-1 (MCP-1/CCL2), Macrophage Inflammatory Protein-1α (MIP-1α/CCL3)) in relation to PDA. **Conclusions**: Even if research shows a strong correlation between specific biomarkers and echocardiographic parameters in patients with PDA, clinical judgment must take these evaluations into account, particularly when determining whether to treat a PDA. Future research should focus on investigating new biomarkers associated with the underlying mechanisms of perinatal ductus arteriosus dynamics in preterm infants.

## 1. Introduction

Patent ductus arteriosus (PDA) in preterm infants presents a significant challenge in neonatal care, marked by ongoing debates about its definition, diagnosis, treatment options, and effects on patient outcomes [1]. The ductus arteriosus (DA) is a fetal structure that connects the main pulmonary artery to the proximal descending aorta, allowing blood to bypass the nonfunctional fetal lung [2,3]. The patency of the fetal DA is essential for fetal survival and is primarily maintained by vasodilatory mechanisms. Relative intrauterine hypoxia [3], along with the activation of Prostaglandin E2 (PGE2) through the EP4 receptor in DA endothelial cells [4,5], plays a crucial role in maintaining fetal ductal permeability. Mediators such as adenosine and atrial natriuretic peptides contribute by upregulating cAMP and cGMP signaling pathways, respectively [6]. The production of nitric oxide (NO) in the endothelium of both the lumen and the vasa vasorum, combined with the formation of carbon monoxide (CO), supports the maintenance of fetal ductal patency. Carbon monoxide inhibits the oxygen-sensing cytochrome P450 and reduces the synthesis of endothelin-1 (ET-1), a potent endogenous vasoconstrictor [7].

Conversely, the initiation of breathing at birth increases blood oxygen levels and decreases PGE2 levels after placental removal, leading to the spontaneous closure of the ductus arteriosus (DA) in full-term infants within 24 to 36 h [8]. Increased oxygen levels enhance oxidative phosphorylation, inhibit potassium channels, and promote Ca^2+^ influx, leading to the vasoconstriction of the DA [9]. Oxygen-induced vasoconstriction is also linked to increased ET-1 synthesis and the production of reactive oxygen species (ROS), which drive the formation of peroxidation products (isoprostanes) in response to oxidative stress [8,10,11]. The permanent anatomical closure of the DA involves a complex remodeling process that transforms it into the ligamentum arteriosum [5,12].

Timing issues related to the closure of the DA include intrauterine obstruction and prolonged patency. Intrauterine closure of the DA is a rare condition that can have severe consequences. Physiologically, excessive blood flow in the fetal pulmonary circulation can lead to severe pulmonary hypertension, right heart failure, fetal hydrops, and even intrauterine death in extreme cases [13,14]. The premature closure of the DA is most linked to maternal ingestion of corticosteroids or nonsteroidal anti-inflammatory drugs (aspirin, ibuprofen, diclofenac) [15] or low levels of circulating endogenous prostaglandins. Several case reports indicate that prenatal closure of the DA may happen after maternal administration of acetaminophen/paracetamol [16,17]. Additionally, a maternal diet high in nutrients that contain prostaglandin synthase inhibitors, such as green tea, dark chocolate, or grape juice, may also contribute to the closure of the DA [13,18,19].

Failure of DA closure beyond 48–72 h after birth leads to patent/persistent ductus arteriosus (PDA), affecting 70% of infants born with a gestational age (GA) of less than 28 weeks [20,21]. Patent ductus arteriosus can lead to hemodynamic issues, including pulmonary over-circulation and reduced blood flow to organs like the bowel, kidneys, brain, and heart due to the ductal steal phenomenon.

These changes can lead to severe conditions, including pulmonary hemorrhage, bronchopulmonary dysplasia (BPD), necrotizing enterocolitis (NEC), intraventricular hemorrhage (IVH), retinopathy of prematurity (ROP), and acute kidney injury [22,23].

The concept of hemodynamically significant patent ductus arteriosus (hsPDA), as well as the management of PDA, is widely debated and currently lacks consensus, leading to considerable variations in clinical practice among neonatal centers. The definition of hsPDA is not standardized and is based on clinical severity scores, which include evidence of systemic hypoperfusion, signs of pulmonary over-circulation, and ultrasound criteria. The term hsPDA typically refers to a symptomatic PDA that leads to hemodynamic instability. Clinical findings like heart murmur, hyperdynamic precordium, bounding pulses, and metabolic acidosis are nonspecific and are unreliable indicators of a hsPDA in the first days of life. These clinical signs poorly correlate with echocardiographic measurements and do not predict treatment responses or outcomes [24]. Color Doppler echocardiography includes criteria such as a ductus arteriosus size larger than 1.5 mm, a left atrial-to-aortic diameter (LA/Ao) ratio exceeding 1.4, and retrograde or absent diastolic flow in the descending aorta, celiac trunk, superior mesenteric artery, or cerebral arteries [22,25]. 

Echocardiography performed by pediatric cardiologists or neonatologists with specialized training in functional echocardiography under the supervision of pediatric cardiologists is the gold standard for PDA assessment. These investigations are often difficult to access, particularly in resource-limited settings. However, echocardiographic parameters have limitations due to interobserver variability, may not always be clinically relevant, and should not be exclusively used to define hsPDA [25]. Kluckow et al. demonstrated that echocardiographic markers have low sensitivity and specificity for ductal significance when evaluated independently compared to transductal diameter [26]

Plasma biomarkers, used alone or combined with echocardiography and clinical signs, can provide valuable and more accessible information. They assess mediators related to PDA closure and hemodynamic responses, helping to identify newborns at higher risk for severe conditions [25]. Some of these biomarkers are currently in clinical use, while others remain under investigation.

Echocardiograms are over ten times more expensive than biomarker tests, leading to a growing interest in using biomarkers to diagnose and predict hemodynamic changes in preterm infants with PDA to reduce healthcare costs [27]. The diagnostic accuracy and reliability of biomarkers for hsPDA vary significantly due to factors such as commercial test assays, threshold cutoffs, and patient characteristics like gestational or chronological age and antenatal and postnatal events. While nonspecific, biomarkers can assist in staging PDA, identifying cases that require treatment, monitoring therapies with pharmacological agents or invasive procedures, and predicting complications [28].

**Figure 1:**
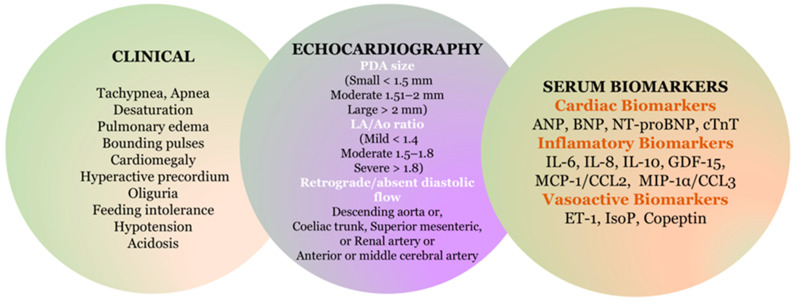
Diagnostics of hsPDA.

Early identification of newborns at risk for delayed closure of the DA is crucial for effective monitoring and treatment, helping to prevent complications related to this condition. (Figure 1).

The ideal management of PDA lacks consensus, posing a challenge due to the rapid changes in hemodynamic status in preterm infants. Indomethacin, ibuprofen, and paracetamol are considered the standard drugs for the treatment of hsPDA. Conservative management and invasive procedures like percutaneous transcatheter device closure or surgical ligation are recommended in documented cases. The purpose of this review was to investigate the relationship between PDA and various plasma biomarkers used to evaluate and diagnose ductal patency during perinatal life, as outlined in the relevant literature.

## 2. Materials and Methods

This narrative review of the literature synthesizes research on biomarker monitoring in preterm infants with PDA. The study includes the most relevant articles concerning enrolled preterm infants and plasma measurements conducted. We searched the National Library of Medicine (MEDLINE)/PubMed and Web of Science for pertinent published studies published up to January 2025, encompassing prospective, retrospective, cohort, and cross-sectional studies, reviews, and meta-analyses. The keywords used in the search were “preterm infant”, “persistent ductus arteriosus”, “patent ductus arteriosus”, “PDA”, “neonatal biomarkers”, “cardiac biomarkers”, and “vasoactive biomarkers”. The “snowball literature searching method” was used to find additional relevant sources from the reference lists of chosen articles. Three reviewers (M.C., E.M., and R.M.) independently assessed the titles and abstracts of the studies identified in the search. We eliminated duplicates and reviewed each abstract for compatibility with the review’s objectives. Any disagreement was resolved through discussion between reviewers.

Out of the 813 identified articles, 728 were excluded because they did not relate to the keyword “biomarkers”. Ultimately, 85 articles were included in our review, and five of these articles were identified through snowballing searching. We noted the author’s name, study type, number of cases, population divisions, gestational age, serum biomarker values in PDA and non-PDA cases, cutoff values, sensitivity, specificity, and key findings for each selected article.

## 3. Results

**Figure 2:**
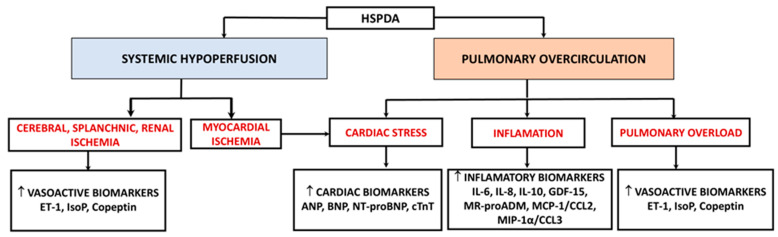
Serum biomarkers in PDA. ↑—increased levels.

Each study used a diverse set of biomarkers for monitoring, with variations in measurement timing and biological sample types. Most assessments focused on blood samples, while urinary assessments were less common. We classified the biomarkers into three categories according to the pathophysiological effects of PDA (Figure 2).

### 3.1. Cardiovascular Markers

#### 3.1.1. Natriuretic Peptides (NPs)

Natriuretic peptides are a family of hormones that directly influence the cardiovascular system, affecting cellular proliferation, angiogenesis, apoptosis, fibrosis, and inflammation [28].

- Atrial natriuretic peptide (ANP)

The atria secrete atrial natriuretic peptide (ANP) in response to increased intracavitary pressure, which typically occurs during volume overload. The physiological effects of ANP primarily occur in the kidneys by dilating the afferent arterioles and constricting the efferent arterioles of the renal tubules, which increases the glomerular filtration rate and promotes diuresis. Additionally, ANP inhibits renin secretion and reduces sodium and water reabsorption, lowering systemic blood pressure [24,29]. Immediately after birth, levels of ANP are significantly higher in newborns compared to older children, with a mean concentration of 227 pg/mL versus 47 pg/mL. This elevation may be attributed to the immature myocardium’s inability to manage the increased left and right ventricular afterload. In preterm infants with hsPDA, even higher ANP levels are observed [30]. Weir et al. found that in preterm infants with hsPDA, median ANP levels decreased from 1240 (201–5483) pg/mL to 266 (62–1108) pg/mL after one course of indomethacin. In comparison, infants with a spontaneously closed ductus arteriosus had a median ANP level of 152 (61–495) pg/mL [31]. Therefore, lower plasma ANP levels may indicate successful therapeutic closure of PDA [30,31].

- Brain natriuretic peptide (BNP)

Ventricular cardiomyocytes release brain natriuretic peptide (BNP) in response to increased wall stress. This peptide helps in regulating blood pressure by promoting vasodilation, diuresis, and sodium excretion, thereby improving cardiac function [32]. Brain natriuretic peptide is the biologically active compound that results from the cleavage at a ratio of 1:1 of the inactive precursor pro-BNP into BNP and the inactive amino-terminal fragment NT-proBNP [33,34]. BNP levels rise immediately after birth, then significantly decrease during the first week of life, eventually reaching adult-specific levels at about one month of age. [35,36]. BNP and pro-BNP are early indicators of myocardial stress, showing similar diagnostic efficacy regardless of the underlying condition. NT-proBNP is considered a better marker for myocardial wall stress than BNP due to its longer half-life of 60–120 min compared to BNP’s 20 min. The serum values of NT-proBNP are approximately six times higher than those of BNP for the same reason [34,37,38].

There is evidence that BNP and NT-proBNP serum levels are influenced by conditions related to prematurity, such as respiratory distress syndrome (RDS), pulmonary hypertension, and BPD [39,40,41], ROP [42,43], sepsis [40,44], and particularly by hsPDA [45,46,47,48,49,50,51,52,53,54,55]. Cucerea et al. observed that surfactant administration was significantly associated (*p* = 0.024) with increased median NT-proBNP levels in a study of 88 preterm infants born at or below 32 weeks of gestational age. The surfactant group had a median level of 12,962.6 (7333.7–25,934.8) pg/mL, while the non-surfactant group had a median level of 9621.6 (3463.2–17,381.8) pg/mL at 24 h of life. Patients in the surfactant group also experienced decreased pre- and post-ductal diastolic pressure, changes that may be related to DA persistence [22].

Various authors have investigated the diagnostic accuracy of BNP and NT-proBNP in relation to hsPDA. The results varied significantly due to differences in commercial testing kits, reference thresholds, study methodologies, definitions of PDA, and the gestational and chronological ages of the patients involved. Thus, the cutoff values for diagnosing hsPDA were established according to these conditions [45,46,47,48,49,50,51,52,53,54,55]. Table 1 and Table 2 represent the main results of studies regarding the association between plasma BNP and NT-proBNP levels and PDA in preterm infants.

Preterm infants with hsPDA exhibited significantly higher mean BNP levels compared to those without PDA (*p* < 0.005) [50,52,55]. Mean BNP levels showed a positive correlation with the echocardiographic assessment of the ductal shunt’s magnitude [45,47,49,50,54,55]. The BNP level on the second day of life was also identified as a predictive factor for PDA closure intervention (AUC of 0.86 and a cutoff of 550 pg/mL) [45].

A study conducted by Mine revealed that preterm infants with hsPDA had elevated levels of BNP that peaked 24 to 48 h after birth compared to those without hsPDA. A BNP level of 250 pg/mL on the second day of life was the most effective cutoff value for predicting the need for indomethacin treatment in hsPDA patients [OR = 5.0 (95% CI, 1.4 to 17.9, *p* = 0.016)]. Additionally, they discovered that a BNP level of 2000 pg/mL five days after birth is the optimal cutoff for predicting the need for surgical ligation [OR = 52.2 (95% CI, 2.1 to 1300, *p* = 0.021)] [51].

Lee et al. investigated the relationship between the magnitude of ductal shunt and BNP levels at 12 and 24 h after birth, finding a stronger correlation at 24 h compared to 12 h of age. They concluded that BNP levels above 900 pg/mL can be used as a guide for early targeted treatment of hsPDA (sensitivity of 54.8%, specificity of 95.2%) [55].

Shin et al. proposed serial BNP measurements during the management of hsPDA as a tool for predicting and diagnosing symptomatic PDA in preterm infants. This approach also serves as a guide for early constrictive responses to cyclooxygenase inhibitors (ibuprofen). They estimated that a BNP level below 600 pg/mL would be used to individually discontinue ibuprofen treatment and avoid unnecessary doses [65]. Hence, we can conclude that BNP values can be used as a clinical tool to monitor the progress of PDA.

NT-proBNP levels correlated with echocardiographic measurements of ductal size and magnitude, similar to [40,47,56]. In contrast, ductal closure was linked to a reduction in NT-proBNP levels [59,62]. This biomarker was also correlated with the presence and severity of an hsPDA [40,61,62], as well as the need for treatment [60,63,64].

Liu et al. established a cutoff value for NT-proBNP at 2331.5 pmol/L on day three, identifying it as the optimal predictive marker for hsPDA. This threshold demonstrated a sensitivity of 97.7% and a specificity of 89.6%, with an AUC of 0.987 (95% CI: 0.973–1.000; *p* < 0.001). The authors suggest testing between 48 and 72 h after birth as the optimal time for assessment [56].

In another study, a cutoff NT-proBNP level of 5900 pg/mL demonstrated 96% sensitivity and 90% specificity for hsPDA, confirming it as an effective predictor with an area under the curve of 0.981 (*p* < 0.001) [63].

König et al. conducted a prospective study involving preterm infants with a gestational age of less than 32 weeks, investigating the specificity of two distinct markers. Serum concentrations of BNP and NT-proBNP were measured prior to the echocardiographic evaluation, which was performed within the first four days of life. Data from fifty-eight enrolled neonates demonstrated that both BNP and NT-proBNP were closely correlated with the size of PDA, making them equally practical for assessing PDA in preterm infants [47].

A review of 34 studies—13 on BNP in 768 infants and 21 on NT-proBNP involving 1459 infants—assessed the accuracy of both biomarkers in diagnosing hsPDA in preterm infants. Despite low-certainty evidence and moderate accuracy, these markers can be considered for initiating and monitoring treatment if validated locally alongside clinical and echocardiographic criteria, even without universal agreements on their use. Testing infants under 30 weeks of gestational age in the first 1 to 3 days of life improves diagnostic accuracy [66,67].

#### 3.1.2. Cardiac Troponin T (cT)

Cardiac troponins (cT) are proteins in the troponin-tropomyosin complex of the myocardium. They facilitate the interaction between actin and myosin in cardiac muscle. They include troponins C (cTnC, calcium-binding), I (cTnI, inhibitory), and T (cTnT, tropomyosin binding), along with tropomyosin [68,69]. Under normal physiological conditions, cardiac troponin T (cTnT) can be detected in plasma at very low levels. Elevated levels indicate myocardial injury and are specific markers for conditions like acute coronary syndromes, myocardial infarction, heart failure, tachyarrhythmias, pulmonary embolism, and sepsis. Troponin levels are detectable in the blood 2 to 4 h after injury, peaking at 12 h and staying elevated for 7 to 10 days [70]. Low levels of cardiac troponin T can occur in chronic cardiac conditions, such as heart failure, as well as in non-cardiac conditions like chemotherapy. The latest high-sensitivity cardiac troponin (hs-cTnT) assays can detect even minor myocardial injury in asymptomatic patients [71].

Limited reports exist on serum troponin levels in newborns, particularly in managing PDA. In a study of 158 full-term newborns, Karlén et al. found that hs-cTnT levels in cord blood [34 (26–44) pg/mL] were elevated compared to adult values and increased further during the first 2 to 5 days of life [92 (54–158) pg/mL]. A plausible reason is cardiac stress resulting from significant changes in the right ventricle, along with pulmonary and systemic vascular resistance during the transition to extrauterine life [69]. A study by Tarkowska et al. found that cTnT levels in newborns correlate with postmenstrual age rather than chronological age. Additionally, these levels are unaffected by gender, delivery method, or blood oxygen saturation [62,68].

Studies have shown elevated serum cTnT levels in neonates with respiratory distress [67,68,72,73] and perinatal asphyxia [69,70,74,75]. Few studies have explored the relationship between cTnT and PDA in preterm infants. Diastolic steal decreases coronary blood flow, causing potential ischemia and alterations in cTnT levels [61,76,77,78]. Table 3 represents the main results of studies regarding cTnT levels and PDA in preterm infants.

Study results vary based on the cTnT assay, methodology, and the gestational and postnatal ages of the infants involved. Research shows a correlation between serum cTnT levels and the size of the patent ductus arteriosus as measured by echocardiography. This indicates that cTnT, in conjunction with clinical evaluation and echocardiography, is a reliable diagnostic tool for PDA. EL-Khuffash et al. demonstrated that cTnT may be a valuable marker of ductal significance and treatment response due to its correlation with echocardiographic markers (ductal diameter, left atrial-to-aortic diameter ratio, and descending aortic end-diastolic velocity) of PDA [(AUC of 0.78 (95% CI 0.66 to 0.90; *p* < 0.001))]. At 48 h of age, the median cTnT level was significantly higher in preterm infants with PDA compared to those with spontaneously closed ductus arteriosus (430 vs. 130 pg/mL; *p* < 0.001) or pharmacologic closure (100 pg/mL; *p* < 0.001) [76]. Tavakoli et al. monitored serial serum troponin levels in 26 patients with PDA and found a declining trend following successful PDA closure (170 ± 160 vs. 70 ± 40 pg/dL; *p* = 0.001) [81]. In prospective research including 60 preterm infants <34 weeks of gestation, the conclusion was that high-sensitivity TnT can significantly detect hsPDA in preterm infants with high sensitivity (93.33%) and specificity (90%) [78].

### 3.2. Vasoactive Biomarkers

Little is known about the role of vasoactive biomarkers in very preterm neonates. Studies have hypothesized significant changes related to various neonatal conditions, including PDA. Although research on vasoactive biomarkers in newborns is limited, it shows that they are valuable in diagnosing infections and cardiovascular conditions. Current tests use nonspecific biomarkers, making identifying the underlying pathophysiology of PDA and targeting interventions effectively unlikely for now.

#### 3.2.1. Mid-Regional Pro-Adrenomedullin (MR-proADM)

Mid-regional pro-adrenomedullin (MR-proADM) is a new biomarker that serves as a precursor to adrenomedullin (ADM), an unstable vasoactive peptide with a half-life of about 22 min, produced by vascular endothelial cells. Factors such as pro-inflammatory cytokines, bacterial endotoxin, hypervolemia, and hypoxia lead to an increase in this biomarker, which is associated with organ dysfunction [82].

MR-proADM demonstrates better stability, allowing for accurate measurement. Due to its immunomodulatory, diuretic, bactericidal, and vasodilatory properties, it has clinical applications in cardiovascular disorders, sepsis, renal failure, tumor pathology, and other conditions involving vascular damage. It indicates the endothelial function, providing data on vascular bed reactivity and coagulation status [83]. Elevated levels of MR-proADM are associated with increased microvascular permeability and plasma leakage into the extracellular space. In patients experiencing septic shock, MR-proADM levels are elevated, leading to hypotension by affecting vascular tone.

MR-proADM is a more reliable marker than procalcitonin (PCT) or C-reactive protein (CRP) for assessing prognosis and mortality risk in patients with sepsis admitted to intensive care units [84,85]. Fahmey et al. observed that septic newborn infants had significantly higher serum levels of MR-proADM, measuring 14.39 ± 0.75 nmol/L, compared to non-septic newborns, who had levels of 3.12 ± 0.23 nmol/L. The study identified a cutoff value for pro-ADM at 4.3 nmol/L, demonstrating a sensitivity of 93.3% and a specificity of 86.7% [86].

Birth weight and gestational age were inversely related to MR-proADM plasmatic levels in the venous umbilical cord (GA 24–31 weeks: 1.4 nmol/L; GA 32–36 weeks: 1.1 nmol/L; GA 37–41 weeks: 1.0 nmol/L) in a prospective study conducted by Admaty on 328 newborn infants. They suggested that in very preterm infants, high MR-proADM plasma levels within 2 to 3 days of life were associated with low blood pressure and diastolic run-off through a PDA [87]. Wu et al. reported significantly reduced plasma MR-proADM levels at day 3 and month 3 after transcatheter closure of PDA [88].

#### 3.2.2. Endothelin-1 (ET-1)

Endothelin-1 is a potent endogenous peptide produced by endothelial cells that acts as both a vasoconstrictor and a bronchoconstrictor. It stimulates natriuresis and diuresis and exerts its effects through two distinct receptor subtypes, triggering pro-inflammatory pathways, increasing superoxide anion production, and facilitating the release of endogenous cytokines. There is a clear correlation between plasma endothelin-1 levels and mortality rates in patients with septic shock, like MR-proADM [89,90].

C-terminal proendothelin-1 (CT-proET-1) is the stable circulating precursor of the active ET-1 molecule. This acts through two G protein-coupled receptors, the endothelin A receptor (ETA) and the endothelin B receptor (ETB). Both receptors induce an increase in intracellular calcium levels. ETA receptors mediate arterial vasoconstriction, while ETB receptors mediate effects on the venous system [85,91].

Research links preterm birth and elevated ET-1 levels to chronic lung disease and pulmonary hypertension in infants [92,93,94]. CT-proET1 is also involved in enabling crucial circulatory adaptations during the transition from fetal to neonatal life.

Letzner [95] identified a correlation between CT-proET-1 levels in treated and untreated PDA, reporting values of 388 (272–723) pmol/L for treated PDA and 303 (152–422) pmol/L for untreated PDA, with a statistically significant *p*-value of 0.011. This finding highlights the potential of CT-proET-1 as a predictor for PDA intervention, particularly when considering the left atrium to aorta (LA/Ao) ratio. In contrast, Grass [96] and Sellmer [97] contended that CT-proET-1 is not a dependable biomarker for assessing the size of PDA or the LA: Ao ratio in very preterm neonates.

#### 3.2.3. Copeptin

Copeptin is the carboxyl-terminal part of the arginine vasopressin (AVP) precursor, synthesized in the hypothalamus. AVP, known as the antidiuretic hormone, has peripheral functions like vasoconstriction, kidney water reabsorption, and central effects. Consequently, antidiuretic hormones are essential for energy homeostasis and dietary habits, making them potential targets in treating metabolic diseases [98].

Unlike peripheral arterioles, AVP decreases resistance in the pulmonary artery, triggering the release of nitric oxide (NO) from endothelial cells, which has a vasodilatory effect during the transition from placental to lung breathing [99].

Copeptin is a stable compound that serves as a biomarker for vasopressin synthesis and functions in conditions like diabetes mellitus, inappropriate antidiuretic hormone secretion, stroke, and various cardiovascular, renal, and pulmonary disorders [100].

Copeptin concentrations were determined by 3 days of life in 167 preterm infants in a study conducted by Benzing. The study found significantly higher levels of copeptin in hsPDA than in closed PDA [38 (8 –199) pmol/L vs. 18 (1–64) pmol/L; *p* = 0.001]. However, elevated copeptin concentrations on day 3 were associated with factors such as invasive respiratory support [35 (19–199) pmol/L], early antibiotic administration [24 (1–199) pmol/L], and confirmed early-onset sepsis [42 (12–64) pmol/L)] [101].

A recent study investigated the relationship between five biomarkers (MR-proADM, NT-proBNP, mid-regional pro-atrial natriuretic peptide (MR-proANP), and C-terminal pro-endothelin-1 (CT-proET1)), and copeptin in correlation with echocardiographic findings of PDA in 139 preterm infants with a GA of less than 32 weeks. On day three of life, levels of MR-proADM, NT-proBNP, MR-proANP, and copeptin were higher in neonates with significant PDA compared to those without. MR-proADM levels were 20% higher in neonates with a significant PDA on days 3 and 6, and there was a correlation between MR-proud and the left atrium to aorta (LA: Ao) ratio [97].

#### 3.2.4. Isoprostanes (IPs)

Reactive oxygen species (ROS) generated in response to oxidative stress can lead to the peroxidation of membrane arachidonic acid, significantly impacting cellular function. Isoprostanes (IsoPs—F2-Isoprostanes) are metabolites formed from peroxidation reactions and can be detected in plasma and urine. Hyperoxia, inflammation, and infection elevate IsoP production [102,103]. Newborns, particularly preterm infants, have higher plasma levels of F2-isoprostanes than healthy adults, primarily due to their limited antioxidant defenses [104]. F2-isoprostanes are biomarkers of oxidative stress associated with perinatal conditions such as intrauterine growth restriction, hypoxic-ischemic encephalopathy, bronchopulmonary dysplasia, periventricular leukomalacia, and retinopathy [97,98,99,100,103,104,105,106]. During the neonatal period, F2-isoprostanes play a physiological role in regulating the patency of the ductus arteriosus, with effects that vary depending on gestational age for both term and preterm infants [107]. Isoprostanes can cause either constriction or dilation of the ductus arteriosus, depending on the balance between thromboxane A2 (TxA2) and EP4 receptors found in ductal endothelial cells. IsoPs cause DA constriction after oxygen exposure by activating the thromboxane A2 (TxA2) receptor, or they can induce vasodilation by activating the prostaglandin E2 receptor 4 (EP4) [103,107]. In preterm DA, the TxA2 receptor expression is low, resulting in reduced contractile capacity, while the EP4 receptor is highly expressed, which promotes dilation. As gestation progresses, TxA2 and its contractile effects become more prevalent [107].

Fifty-three preterm infants born at or before 32 weeks of gestation participated in Inayat’s study, which evaluated antioxidants and oxidative stress biomarkers related to PDA using blood and urine samples collected within 24 to 48 h after birth. At 24 and 48 h, plasma 8-isoprostane (8-isoPGF2α) levels were significantly lower in preterm infants who subsequently developed a hsPDA (6060.9 ± 5302.5 pg/mL, *p* < 0.01) than in those who did not (13,281.5 ± 9161.7 pg/mL). The urinary levels of 8-isoprostane were similar in both the hsPDA group and infants without PDA, showing no change in response to treatment within the hsPDA group. The authors considered that preterm infants exhibit low levels of plasma and urinary isoprostanes shortly after birth due to relative hypoxia, suggesting that 8-isoprostane could serve as a biomarker for hsPDA [108].

Coviello et al. studied the correlation between urinary isoprostane (IsoP) levels and hsPDA in sixty preterm infants (GA 23 to 34 weeks) diagnosed with RDS. The results indicated significantly higher IsoPs levels in infants with ibuprofen-treated hsPDA and who required surgical closure compared to those without PDA on the second day of life [2700.0 (1205.7–6688.0), 5028.7 (1233.0–17,770.0)] vs. 969.9 (541.0–1470.6) ng/mg of creatinine; *p* < 0.01]. On the 10th day of life, urinary IsoPs levels were comparable in infants with and without hsPDA. The authors revealed a strong predictive ability of urinary IPos levels on the second day of life regarding the risk of developing hsPDA (AUC 0.78; 95% CI 0.65–0.71, *p* < 0.0001). They identified a cutoff level of 1627 ng/mg of creatinine, which predicts hsPDA with an 82% sensitivity and a 73% specificity [109]. It seems that urinary IsoPs is a promising non-invasive biomarker for the prediction of hsPDA if an early (in the first 48 h of life) rapid dosing method is developed.

### 3.3. Inflammatory Biomarkers

Pro-inflammatory conditions are known to delay the postpartum closure of the ductus arteriosus. Prenatal and postnatal inflammation significantly contribute to PDA, causing increased vascular tone and delayed closure. Chorioamnionitis triggers vascular remodeling via pro-inflammatory cytokines like interleukin-1 and TNF-alpha (tumor necrosis factor), resulting in PDA and contributing to persistent pulmonary hypertension in newborns. Elevated levels of interleukins (IL-6, IL-8, and IL-12) have been documented in cases of pulmonary diseases and vascular remodeling. The administration of antenatal steroids and anti-inflammatory medications for treating chorioamnionitis reduces both the risk and severity of PDA [110].

The ambiguity between inflammatory and infectious processes has complicated accurate assessments in studies. The biomarkers evaluated were not specific for identifying PDA, as they could also be elevated in other conditions within the same age group.

#### 3.3.1. Interleukin-6 (IL-6)

Interleukin-6 (IL-6) is a cytokine that plays a key role in regulating the immune response and acute-phase reactions. IL-6 promotes the increase of IgM, IgG, and IgA and stimulates T helper cell proliferation during inflammation or infection. Although IL-6 is a promising biomarker for diagnosing certain conditions, its effectiveness can vary based on the context. There is significant individual variation in IL-6 levels. Regarding gestational age, IL-6 levels are higher in preterm infants compared to full-term newborns. The determination of IL-6 from umbilical cord blood has a sensitivity of over 87% for early-onset sepsis [111,112]. Serological tests have a sensitivity that ranges from 75% to 85%. The cutoff levels for these tests are set at 80 pg/mL for the first day of life, 40 pg/mL for days 2 to 7, and 30 pg/mL after the first week. The specificity of these tests is relatively good, ranging from 72.8% to 88% [108]. Additionally, interleukin-6 (IL-6) plays a significant role in increasing vasodilatory prostaglandins, which can contribute to PDA [111].

#### 3.3.2. Interleukin-8 (IL-8)

Interleukin-8 (IL-8) is a pro-inflammatory cytokine primarily produced by monocytes, essential for host defense against infectious diseases. It regulates inflammatory and immune responses and serves as a crucial chemotactic factor, facilitating neutrophil recruitment and activation [113]. IL-8 can be used as an early marker for the early diagnosis of neonatal sepsis [114,115]. A level of 60 pg/mL was the upper limit for IL-8 in non-infected neonates, while a level of 142.4 ± 111.6 pg/mL was found in newborns with early-onset sepsis [116]. This cytokine is linked to the persistence of the DA and the evaluation of response to closure. This cytokine is associated with DA persistence and the reaction to its closure, involving a complex interaction that is still poorly understood [110,117].

#### 3.3.3. Interleukin-10 (IL-10)

Interleukin-10 (IL-10) is a cytokine involved in maintaining systemic homeostasis and modulating inflammation. IL-10 is produced by various lymphoid, myeloid, and mast cells and belongs to the IL-10 cytokine family, which also includes IL-19, IL-20, IL-22, IL-24, IL-26, and interferons. The ability of interleukin-10 (IL-10) to suppress pro-inflammatory cytokines such as TNF-α, IL-1, and IL-6 makes it a promising therapeutic target for treating inflammatory disorders [118].

A study by Sellmer et al. revealed that newborns with a hsPDA exhibited elevated levels of interleukin-6, interleukin-8, and interleukin-10. In contrast, complement component 8 and carboxypeptidase levels were decreased compared to newborns without persistent fetal circulation [88].

#### 3.3.4. Growth Differentiation Factor 15 (GDF-15)

Growth Differentiation Factor 15 (GDF-15) is associated with inflammatory processes and acts as a stress-responsive cytokine. Higher levels of GDF-15 are associated with an increased risk of chronic kidney disease, cardiovascular diseases, and pulmonary conditions like pulmonary hypertension and pulmonary fibrosis [119].

During pregnancy, the placenta releases increased amounts of GDF-15, leading to higher levels of this protein in maternal serum. Almudarez et al. discovered that GDF-15 levels decreased with gestational age, while elevated levels were linked to respiratory issues, more extended hospital stays, and increased ventilator support [120]. GDF-15 could be a valuable biomarker for monitoring children with congenital heart disease and congestive heart failure, helping assess disease severity and guide treatment [121].

#### 3.3.5. Monocyte Chemoattractant Protein-1 (MCP-1/CCL2)

Monocyte chemoattractant protein-1 (MCP-1/CCL2) is a cytokine from the chemokine family that acts as a strong attractant for monocytes by activating G protein-coupled receptors. It plays a key role in the migration and infiltration of monocytes and macrophages. This migration across the vascular endothelium is typically a physiologic process for monitoring tissues, but it can also occur in response to inflammation during pathological conditions. Experimental evidence indicates that CCL2 deficiency is linked to a significant decrease in arterial lipid deposits, while elevated levels of CCL2 are associated with atherosclerosis [122,123].

#### 3.3.6. Macrophage Inflammatory Protein-1α (MIP-1α/CCL3)

Macrophage Inflammatory Protein-1α (MIP-1α/CCL3) is a member of the chemokine family. It can be secreted by various immune cells, including monocytes, T lymphocytes, B lymphocytes, neutrophils, dendritic cells, and natural killer (NK) cells, alongside MIP-1β/CCL4. MIP-1α/CCL3 plays several roles, including recruiting inflammatory cells, inhibiting stem cell functions, and supporting the immune response. Typically, the measured levels of this chemokine are low. Cells that secrete MIP-1α/CCL3 are found in areas experiencing accelerated inflammation or in regions where bone resorption occurs. Patients diagnosed with conditions such as Sjögren’s syndrome, multiple myeloma, or rheumatoid arthritis often exhibit elevated levels of MIP-1α/CCL3. Additionally, patients who have suffered a myocardial infarction or have conditions leading to congestive heart failure also show increased levels of this chemokine [124].

Yu-Jen Wei et al. investigated the association between intrauterine inflammation and PDA in preterm infants. They assessed the fetal inflammatory response by measuring interleukin 6 (IL-6) levels in the umbilical cord. A level above 11 pg/mL suggests a strong inflammatory response, increasing the risk of intraventricular hemorrhage, chronic lung disease, and cerebral palsy [110]. A study conducted by Olsson indicates that elevated levels of interleukin-6 (IL-6), IL-8, IL-10, IL-12, growth differentiation factor 15 (GDF-15), monocyte chemoattractant protein-1 (MCP-1/CCL2), and macrophage inflammatory protein-1α (MIP-1α/CCL3) are associated with PDA [117]. Aikio et al. studied the impact of paracetamol on serum inflammatory biomarkers in very preterm infants with respiratory distress. During the early treatment (<60 h), Paracetamol had no effect on cytokine levels, but later treatment (60–120 h) was associated with lower IL-10 and MIP-1α/CCL3. It is unclear whether the decrease in cytokines results from reduced circulatory stress due to the PDA constriction caused by the treatment or if it reflects a direct systemic anti-inflammatory effect [125].

### 3.4. Importance and Limitation of Biomarkers Use

Biomarkers are essential tools for diagnosing diseases, predicting outcomes, and guiding treatment. They enhance clinical evaluation and therapy monitoring by accurately reflecting changes in physiological and pathological processes. Ideal biomarkers should be reliable, with high sensitivity, specificity, accuracy, and positive predictive value. For broad clinical acceptance, they must provide accurate measurements and timely alerts while remaining minimally invasive, accessible, user-friendly, and cost-effective.

Diagnosing PDA can be challenging due to the limited value of clinical findings and the high cost of echocardiographic evaluations. Studies indicate that serum biomarkers such as BNP, NT-proBNP, and cardiac troponin correlate with echocardiographic parameters, effectively predicting and identifying hsPDA in preterm neonates. These biomarkers can differentiate between hsPDA, non-hsPDA, and no PDA, helping to prevent unnecessary interventions. Established cutoff values for these biomarkers serve as reliable indicators for determining the need for echocardiography and appropriate treatments [45,46,47,48,49,50,51,52,53,54,55,56,57,58,59,60,61,62,63,64,76,77,78,79]. Some vasoactive biomarkers, such as MR-proADM, ET-1, copeptin, and isoprostanes, seem promising, but their lack of validation in larger patient cohorts makes them unlikely to be used alone [88,95,97,109].

Studies indicate that inflammatory biomarkers lack sufficient specificity and sensitivity for routine clinical use due to the influence of antenatal and perinatal factors on their levels. For some biomarkers, essential metrics such as cutoff, specificity, sensitivity, and predictive values for PDA remain undefined. As a result, no reliable predictive inflammatory biomarkers have been identified [110,111,117,125].

Patent DA is a complication of prematurity, sharing multifactorial causes with RDS, BPD, NEC, and ROP. These conditions are interrelated. For instance, there is a bidirectional interaction between RDS and PDA, creating a vicious cycle. Hypoxemia associated with respiratory distress syndrome delays the closure of the DA, while pulmonary hypoxemia further reduces surfactant production. Administering surfactant can lower pulmonary vascular resistance and enhance blood flow through the ductus arteriosus. PDA causes pulmonary over-circulation, decreased pulmonary compliance, a higher need for invasive ventilation, and an increased risk of bronchopulmonary dysplasia. These changes may impact the dynamics and specificity of nonspecific biomarkers in preterm infants. Extensive multicenter prospective trials are needed to address this issue and to validate these biomarkers. Future research on the development of pathway-specific biomarkers and interventions is required, as well as the use of advanced techniques for identifying specific genetic and epigenetic markers for early detection of patients at risk for PDA.

#### Limitation of This Review

This article provides a general overview of the available literature on serum biomarkers and PDA in preterm infants. The literature is considerable and varied, making a structured methodological search challenging. The paper does not use a meta-analytic approach due to the significant variability of selected studies on biomarkers related to PDA, particularly in terms of their methodologies. Clearly, a meta-analysis of key biomarkers, including future discoveries, will be valuable.

## 4. Conclusions

Managing patent ductus arteriosus remains a significant challenge for neonatologists and pediatric cardiologists, with ongoing debate about its definition, the need for treatment, and the most effective options. When considered in isolation, clinical signs, echocardiographic findings, and serum biochemical markers cannot reliably determine the hemodynamic significance of a PDA. Clinical judgment must integrate these evaluations, particularly when deciding to treat a PDA. This thoughtful approach can guide appropriate interventions and help avoid unnecessary treatments, ensuring optimal patient care.

The use of biomarkers in diagnosing and managing PDA is an underexplored opportunity, even if the ability of biomarkers like natriuretic peptides, troponin, and isoprostanes to predict the early diagnosis of hsPDA in preterm infants has been demonstrated in several studies. Some biomarkers have shown promising results, but their clinical application remains unclear. Research indicates a strong correlation between these biomarkers and echocardiographic parameters in patients with significant patent ductus arteriosus, suggesting that serial measurements could help assess clinical outcomes and treatment responses. Because of differences in testing characteristics, timing of evaluation, and infant status, standardizing variability in practice is challenging. Therefore, it is not feasible to recommend specific cutoff values for these biomarkers as universal diagnostic criteria for hsPDA. Threshold cutoffs should be determined based on age-specific normative values that are locally validated for the commercial test used and the preterm infant population. However, biomarkers could be valuable diagnostic tools when combined with clinical data, especially when echocardiography is unavailable or in low-resource areas. Biomarkers are cost-effective, easily accessible, and can improve the information obtained from other diagnostic methods.

Future long-term randomized controlled trials should focus on investigating new biomarkers associated with the underlying mechanisms of perinatal ductus arteriosus dynamics in preterm infants to increase detection sensitivity and economic feasibility. Furthermore, developing a composite scoring system that incorporates biomarker values specific to gestational and chronological ages, in addition to clinical parameters, could enhance diagnostic accuracy and applicability for personalized precision treatment. Artificial intelligence could also improve the accuracy of PDA diagnosis and prognosis through multifactor analysis in machine learning models for a personalized treatment approach. 

## Figures and Tables

**Table 1:** Summary of associations between plasma BNP levels and PDA in preterm infants.

	GA Weeks	n	Age Days	BNP (pg/mL)					
				PDA	No PDA	Cutoff Value	Sensitivity	Specificity	Study Findings
Czernik [45]	<28 Median 26	67	1–2	1069 (564–1845) 87 (17–130) #	247 (121–463)	550	83%	86%	BNP is correlated with DA size (R = 0.46, *p* < 0.001) BNP is predictive for PDA treatment
Cui Q [46]	28–32	67	3	95.20 ± 7.42	70.15 ± 6.44	-	68.9%	69%	BNP is correlated with early diagnosis and progression of PDA
König [47]	<32	58	1–4	486.5 (219–1316)	190 (95.5–514.5)	-	-	-	BNP is correlated with PDA size (R = 0.35, *p* = 0.0066)
Parra-Bravo [48]	< 32	29	3–5	1061.9 ± 105.7	219.9 ± 227.8	486.5	81%	92%	BNP is correlated with hsPDA (R = 0.71; *p* < 0.001)
Kim [49]	<37 32.7 (28.4–35.8)	28	4	654.68 (428.29–1280)	124.52 (37.21–290.49)	412	100%	95%	BNP is correlated with hsPDA
Choi [50]	25–34	66	3	2896 ± 1627	208 ± 313	1110	100%	95.3%	BNP is correlated with the magnitude of the DA shunt
Mine [51]	<33	46	2–3	283.4 (123.1–226.2)	88.4 (38.6–191.4)	250 2000	80%	40%	BNP is predictive for PDA treatment (indomethacin) BNP is predictive for PDA surgery
Sanjeev [52]	≤34	29	2–28	508.5 ± 618.2	59.5 ± 69.9	70	92.9%	73.3%	BNP is correlated with hsPDA
Kalra [53]	<34	52	3–7	2410 (420–2770)	23.6 (13.1–32.8)	123	100%	100%	BNP is predictive for decision for treatment
Zekri [54]	≤35	73	1–2	536 (36–5665)	59.25 (11.5–331)	160.5	80.49%	90.62%	BNP is correlated with PDA size
Lee [55]	27.1 ± 2.2	73	1	921 (318–2133)	152 (91–450)	>200 >900	83.9% 54.8%	61.9% 95.2%	BNP at 24 h is correlated with the magnitude of the of the DA shunt BNP at 24 h—guide for early targeted treatment of hsPDA

Data are presented as mean ± SD or median (range) due to lack of Gaussian distribution; GA—gestational age; n—number of cases; DA—ductus arteriosus; PDA—patent ductus arteriosus; BNP—brain natriuretic peptide; hsPDA—hemodynamically significant patent ductus arteriosus; # after intervention.

**Table 2:** Summary of associations between plasma NT-proBNP levels and PDA in preterm infants.

	GA Weeks	n	Age Days	NT-proBNP (pg/mL)					
				PDA	No PDA	Cutoff Value	Sensitivity	Specificity	Study Findings
Liu Y [56]	30.6 ± 1.5	120	1 2 3	2050.0 ± 590.5 5716.8 ± 2267.0 5505.1 ± 2210.2	1865.4 ± 436.6 2765.5 ± 793.1 1618.7 ± 782.3	3689 2331.5	83.7% 97.7%	93.5% 89.6%	NT-proBNP is predictive for hsPDA NT-proBNP is correlated with the magnitude of DA shunt Day three of life is the optimal testing time
Nuntnarumit [57]	<37	35	2	16,353 (10,316–104,998)	3914 (1535–19,516)	10,180	100%	91%	NT-proBNP is predictive for HsPDA
Fritz [40]	≤31	118	1–7	7843 (2915–14,116)	1896 (1277–5200)	-	-	-	NT-proBNP is correlated with the severity of PDA
König [47]	<32	58	1–4	10,858.5 (6319–42 108)	7488 (3363–14 227.5)	-	-	-	NT-proBNP is correlated with PDA size
Harris [58]	< 30	51	3	1840 (1058)	178 (140)	287	92%	92%	NT-proBNP is predictive for hsPDA
Gudmundsdottir [59]	<28	98	3	14,600 (7740–28,100) 32,300 (29,100–35,000) *	1810 (1760–6000)	6001–9000 15,001–18,000	61% 66%	20% 66%	NT-proBNP is predictive for spontaneous DA closure Predictive for PDA surgery
Ramakrishnan [60]	29	56	2	6952	1206	2850	90%	89%	NT-proBNP is predictive for PDA treatment
Asrani [61]	<34	70	1–5	18,181.02	3149.23	3460	88%	72%	NT-proBNP is an excellent diagnostic test for PDA
Rodriguez-Blanco [62]	≤32	85	2–3	33,171 (5337–60,684)	2065 (1093–4448)	5099	94%	82%	NT-proBNP at 48–96 h of life can be used to exclude hsPDA
Buddhe [63]	27 ± 2.6	69	3–5	24,420 ± 3190	3072 ± 332	5900	96%	90%	NT-proBNP helps timing of intervention of a hsPDA
Lin [64]	30.8 ± 3.3	36	2	9233.5	4262.5	-	-	-	NT-proBNP might predict the effectiveness of the treatment

Data are presented as mean ± SD or median (range) due to lack of Gaussian distribution; GA—gestational age; n—number of cases; PDA—patent ductus arteriosus; NT-proBNP—N-terminal pro-brain natriuretic peptide; hsPDA—hemodynamically significant patent ductus arteriosus. * Need for surgery.

**Table 3:** Summary of associations between plasma cTnT levels and PDA in preterm infants.

	GA Weeks	n	Age Days	cTnT (pg/mL)					
				PDA	No PDA	Cutoff Value	Sensitivity	Specificity	Study Findings
Asrani [61]	<34	70	2	251.5 ± 65.6	161 ± 22.4	170	70%	55%	cTnT is a fair diagnostic test for PDA
EL-Khuffash [76]	28 (26.1–29.5)	80	½–2	430	130	200	70%	75%	cTnT significantly correlated with echocardiographic markers of DA significance
Mohamed [77]	31.7 ± 61.57	77	2;5–7	310 ± 60	160 ± 30	-	-	-	cTnT is correlated with PDA size
Omar [78]	<34	60	1–4	182.7 ± 59.62	67.23 ± 25.96	>100	93.33%	90%	cTnT can detect hsPDA
Vaisbourd [79]	<32	43	1–3	hsPDA 200 ± 100 nhsPDA 120 ± 100	100 ± 100	-	-	-	cTnT is as sensitive as echocardiographic findings in hsPDA
Veysizadeh [80]	32.658 ± 1.554	36	1–3	124.506 ± 113.138	112.275 ± 66.546	-	-	-	There is no correlation between PDA and cTnT

Data are presented as mean ± SD or median (range) due to lack of Gaussian distribution; GA—gestational age; n—number of cases; PDA—patent ductus arteriosus; cTnT—Troponin; hsPDA—hemodynamically significant patent ductus arteriosus; nhsPDA—Nonsignificant PDA.

## Data Availability

The data presented in this study are available on request from the corresponding author.
